# Can Metformin Exert as an Active Drug on Endothelial Dysfunction in Diabetic Subjects?

**DOI:** 10.3390/biomedicines9010003

**Published:** 2020-12-22

**Authors:** Teresa Salvatore, Pia Clara Pafundi, Raffaele Galiero, Luca Rinaldi, Alfredo Caturano, Erica Vetrano, Concetta Aprea, Gaetana Albanese, Anna Di Martino, Carmen Ricozzi, Simona Imbriani, Ferdinando Carlo Sasso

**Affiliations:** 1 Department of Precision Medicine, University of Campania Luigi Vanvitelli, Via De Crecchio 7, I-80138 Naples, Italy; teresa.salvatore@unicampania.it; 2 Department of Advanced Medical and Surgical Sciences, University of Campania Luigi Vanvitelli, Piazza Luigi Miraglia 2, I-80138 Naples, Italy; piaclara.pafundi@unicampania.it (P.C.P.); raffaele.galiero@unicampania.it (R.G.); luca.rinaldi@unicampania.it (L.R.); alfredo.caturano@unicampania.it (A.C.); erica.vetrano@gmail.com (E.V.); concetta.aprea27@outlook.it (C.A.); gaetanaalbanese@hotmail.it (G.A.); annadimarti@alice.it (A.D.M.); carmenricozzi28@gmail.com (C.R.); simo.imbriani@gmail.com (S.I.)

**Keywords:** metformin, endothelial dysfunction, diabetes, CV risk

## Abstract

Cardiovascular mortality is a major cause of death among in type 2 diabetes (T2DM). Endothelial dysfunction (ED) is a well-known important risk factor for the development of diabetes cardiovascular complications. Therefore, the prevention of diabetic macroangiopathies by preserving endothelial function represents a major therapeutic concern for all National Health Systems. Several complex mechanisms support ED in diabetic patients, frequently cross-talking each other: uncoupling of eNOS with impaired endothelium-dependent vascular response, increased ROS production, mitochondrial dysfunction, activation of polyol pathway, generation of advanced glycation end-products (AGEs), activation of protein kinase C (PKC), endothelial inflammation, endothelial apoptosis and senescence, and dysregulation of microRNAs (miRNAs). Metformin is a milestone in T2DM treatment. To date, according to most recent EASD/ADA guidelines, it still represents the first-choice drug in these patients. Intriguingly, several extraglycemic effects of metformin have been recently observed, among which large preclinical and clinical evidence support metformin’s efficacy against ED in T2DM. Metformin seems effective thanks to its favorable action on all the aforementioned pathophysiological ED mechanisms. AMPK pharmacological activation plays a key role, with metformin inhibiting inflammation and improving ED. Therefore, aim of this review is to assess metformin’s beneficial effects on endothelial dysfunction in T2DM, which could preempt development of atherosclerosis.

## 1. Introduction

Type 2 diabetes mellitus (T2DM) has been recognized for long a disease of the cardiovascular system, so that, at the beginning of the 1980s, someone has established it as a “cardiovascular disease diagnosed by glycemia”. This anecdotal definition preempted the scientific evidence produced by Haffner in 1998, who demonstrated a similar mortality risk from Coronary Heart Disease (CHD) in a diabetic patient without previous myocardial infarction (MI) as compared to a non-diabetic subject who had suffered from a previous heart ischemic accident [1].

Metformin is the drug of choice for T2DM treatment and most of patients are usually treated first with this drug, and then with other anti-hyperglycemic agents in add-on to their therapeutic regimen as required [2]. Large evidence from the literature, both from preclinical and clinical studies, strengthen the anti-atherosclerotic properties of this drug [3].

In this review, we address the impact of metformin’s administration on macrovascular complications of diabetes. In particular, we analyze metformin’s beneficial effects on that distinctive pathophysiological condition named endothelial dysfunction (ED), which preempts the early development of atherosclerosis.

### 1.1. Endothelial Function

The endothelium is a thin monolayer covering the inner surface of blood vessels. It represents a barrier between circulating blood and all tissues and secretes a plethora of bioactive mediators of vascular tone [4].

The most important factor for the maintenance of vascular homeostasis is nitric oxide (NO), derived from the oxidation of L-arginine in the catalysis of endothelial nitric oxide synthase (eNOS), an enzyme constitutively expressed in endothelial cells (ECs) [5]. Once produced, NO rapidly moves to vascular smooth muscle cells (VSMCs), where it activates the soluble guanylate cyclase system which, in turn, increases cyclic guanosine-3′,5 mono phosphate (cGMP) and determines VSMCs relaxation [6]. Other vasodilators released by endothelial cells include endothelium derived hyperpolarization (EDH) factor of VSMCs, prostacyclin I_2_ (PGI_2_), bradykinin, histamine, serotonin, and substance P. As opposed to vasodilators, endothelium secretes a number of vasoconstrictors, especially cyclooxygenase-derived prostanoids, endothelin-1 (ET-1), angiotensin II (ANG II) and reactive oxygen species (ROS), usually associated with ED [7,8]. The relative contribution of vasodilator signals to the endothelium-dependent relaxation depends on blood vessels size, with NO dominant in conduit arteries and EDH factor as the diameter of the arteries decreases [9].

The fine equilibrium between these opposite factors is crucial to maintain a normal arterial patency. Conversely, an unbalanced production in favor of vasoconstrictor signals compromises the vascular auto-regulation and the functional and structural integrity of the endothelium, thus originating the ED [7].

### 1.2. Endothelial Dysfunction

ED is a condition of altered metabolism and function of endothelium inducing vascular injury and defective repair. Functionally, ED can be defined as a reduced bioavailability of NO, which affects the impaired response to an endothelium-dependent vasodilator such as acetylcholine.

Endothelium-derived NO not only keeps blood flow, though it also acts as a negative modulator of platelet aggregation, pro-inflammatory gene expression, ICAM-1 (intercellular adhesion molecule 1) and VCAM-1 (vascular cell adhesion molecule 1) production, E-selectin expression, ET-1 synthesis, VSMC proliferation, and lipoprotein oxidation [10]. Thus, ED is characterized by a series of features which goes beyond the hemodynamic dysregulation, including excess production of reactive oxygen species (ROS), enhanced expression of adhesion molecules and inflammatory mediators [11], and increased permeability of vascular endothelium. All of these promote both beginning and progression of atherogenesis [12,13].

The predictive role of ED on the cardiovascular risk has been largely documented in clinical studies by non-invasive, semi-invasive and invasive techniques measuring ED in humans in situ [14,15]. Besides ED functional measures, circulating levels of adhesion molecules and proinflammatory cytokines have also been used as surrogate markers of endothelial activation and cardiovascular risk [16].

## 2. Endothelial Dysfunction in Diabetes

The literature extensively supports ED as an important risk factor for the development of T2DM cardiovascular complications [17].

Based on a state of insulin resistance (IR), the interaction of three pathological conditions frequently associated with diabetes (hypertension, dyslipidemia and hyperglycemia), plays a pivotal role in the pathogenesis of the atherosclerotic process [18]. In this scenario hyperglycemia, playing a key role in any complication of diabetes [19], is a leading actor.

Acute hyperglycemia, achieved by intra-arterial infusion of dextrose, has been documented to impair endothelium-dependent vasodilation in healthy humans [20]. Likewise, the acute increase in plasma glucose after administration of oral glucose tolerance test (OGTT) determines, within a 1–2 h time period, a reduction of flow-mediated vasodilation in non-diabetic subjects, with a higher response in individuals with impaired glucose tolerance (IGT), and even more in those with diabetes [21]. A similar harmful effect is likely expected from prolonged and repeated post-prandial hyperglycemias, as it may routinely happen in T2DM. These hyperglycemic spikes may exert a dramatic and long-lasting epigenetic “memory” effect on the endothelial function, as reported in ECs cultured in high glucose and then restored to normoglycemia [22], which suggests transient hyperglycemia as a potential HbA1c–independent risk factor for diabetic complications [23]. A recent study in small mesenteric arteries from healthy and diabetic *db*/*db* mice has demonstrated that both acute and chronic exposure to high glucose interfere with local and conducted vasodilation in the resistance vasculature mediated by EDH [24].

**Figure 1:**
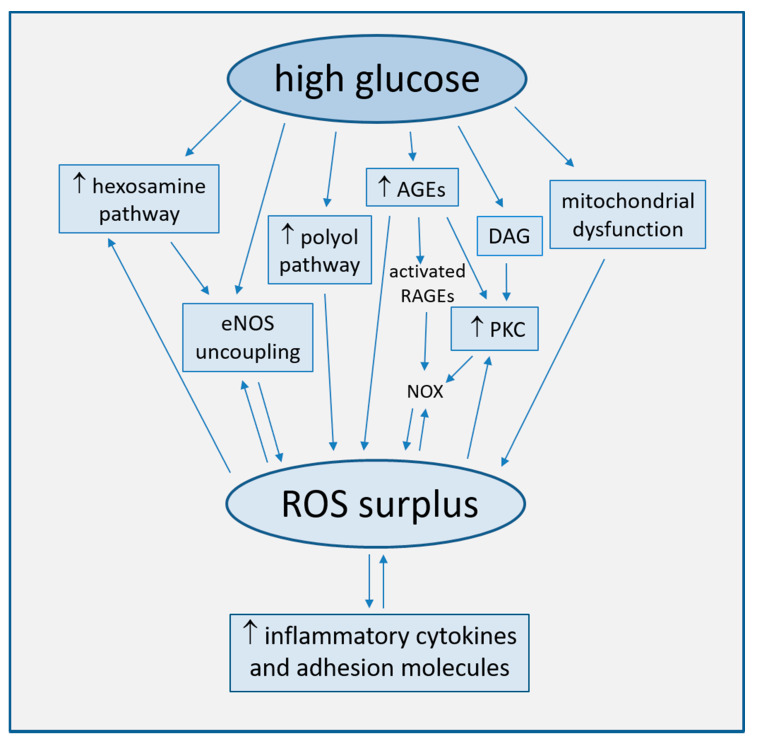
Main mechanisms of high glucose-induced endothelial dysfunction (direct arrows indicate the direction of the pathway, whilst double arrow stands for bidirectional pathway).

Strong accumulating evidence suggests oxidative stress, defined as increased formation of ROS, reactive nitrogen species (RNS), and/or decreased antioxidant potentials, as the cornerstone of ED in the development of diabetic complications [25]. This condition triggers the production of pro-inflammatory cytokines and adhesion molecules responsible of intimal lesions formation [26,27]. Indirectly, some downstream processes (e.g., insulin resistance, formation of oxidized-low density lipoprotein (ox-LDL), inhibition of AMP-protein kinase (AMPK), and adiponectin) contribute to inflammation during the progression of atherosclerosis [28]. In turn, inflammation enhances ROS production, with a consequent arise of a variety of vicious cycles which intertwine each other, thus featuring the pathogenic complexity of the diabetes-accelerated atherosclerosis [29,30]. Moreover, endothelial damage increases albuminuria, both an independent and strong marker of CV risk [31,32]. The mechanisms by which hyperglycemia induces endothelial dysfunction are summarized in Figure 1 and are described in detail in the following paragraphs.

### 2.1. Increased ROS Production

Oxidative stress plays a major role in the pathophysiology of diabetic vascular disease [28,33]. Such a role is consistent with large evidence that increased concentrations of glucose in cultured endothelial cells induce an overproduction of ROS, with the subsequent activation of intracellular signal transduction pathways leading to ED [34,35]. High glucose concentration has been well established to cause endothelial cell damage by both an overproduction of ROS in mitochondria and by multiple biochemical pathways.

#### 2.1.1. Uncoupling of eNOS

The deep reduction in endothelium-dependent vasodilatation associated with T2DM can be linked to changes in eNOS phosphorylation and desensitization induced by signal transduction pathways activated by ROS surplus. As an example, oxidative stress can activate the hexosamine biosynthetic pathway under diabetic and hyperglycemic conditions. This activation is further accompanied by an increase in O-linked N-acetylglucosamine modification of eNOS and a decrease in O-linked serine phosphorylation at residue 1177 [36].

The functional disturbance of the enzyme results in the production of superoxide anion (O_2_^−^**·**) rather than NO, a phenomenon named eNOS uncoupling [37,38].

The ability of eNOS to generate NO can be disabled by the deficiency of tetrahydrobiopterin (BH4), an essential enzyme co-factor, which transforms eNOS into an oxidant-producing enzyme of O_2_^−^**·** [39,40]. ROS may induce oxidative changes of BH4 to dihydrobiopterin (BH2), a BH4 competing compound ineffective as eNOS co-factor. BH2/BH4 competition results in the dissociation of dimeric eNOS to the monomeric form, which acts through its oxygenase domain as an NADPH oxidase, further enhancing ROS generation, in a harmful perpetuation of a vicious circle [10,41,42,43]. Interestingly, the hyperglycemia-induced ED in normal subjects may be prevented by pre-treatment with the BH4 active isomer, 6R-BH4, whilst not by its inactive stereoisomer, 6S-BH4 [44]. In addition, the oral treatment with sepiapterin, a stable precursor of BH4, reduced oxidative stress and improved acetylcholine-mediated endothelium-dependent vasodilation in small mesenteric resistance arteries from *db*/*db* obese diabetic mice [45].

GTP cyclohydrolase I (GTPCH I) is the first enzyme in the BH4 biosynthetic pathway, constitutively expressed in endothelial cells and critical for the maintenance of NO synthesis [46]. Studies in HUVECs exposed to high glucose and in streptozotocin-injected diabetic mice have found that hyperglycemia may trigger BH4 deficiency by increasing 26S proteasome-mediated degradation of GTPCH I [47]. This degradation could be either prevented or improved by AMPK overexpression or activation [48].

NO derived from dimeric eNOS and O_2_^−^**·** from monomeric eNOS induces the formation of peroxynitrite (ONOO^−^**·**). This may facilitate the release of zinc from the zinc-thiolate cluster of eNOS, which is useful to maintain the dimeric structure of the enzyme, thus resulting in a further enhancement of eNOS uncoupling. Since loss of zinc and eNOS uncoupling activity have been both observed in ECs cells exposed to elevated glucose and in tissues of a diabetic mice model, we may hypothesize a significance of this process under in vivo conditions in diabetes [10,49].

The functions of many proteins may be affected by increased oxidant levels. As an example, a characteristic reaction of ONOO^−^**·** is the nitration of protein-bound tyrosine residues to generate 3-nitrotyrosine–positive proteins [50]. Some researchers have suggested that an increased nitration of PGI_2_ synthase (PGIS), more likely via dysfunctional eNOS, may characterize the diabetic disease. Such a hypothesis stands on observations that exposure of isolated bovine coronary arteries to high glucose switched angiotensin II–stimulated PGI_2_-dependent relaxation into a persistent vasoconstriction [51]. As well, a significant suppression of PGIS activity, along with increased O_2_^−^**·** and PGIS-nitration, was also observed in aortas of streptozotocin-treated diabetic mice [51].

#### 2.1.2. Mitochondrial Dysfunction

The mitochondrial electron transport chain (ETC) is the primary source of hyperglycemia-induced ROS production via a greater oxygen use, increased redox potential and shift of O_2_ transport towards the respiratory chain complex II [25,29]. Other mechanisms of mitochondrial dysfunction include increased NADH/FADH2 ratio [52] and mitochondrial fission, which triggers an accumulation of fragmented mitochondria with impaired ETC activity [53].

#### 2.1.3. Activation of the Polyol Pathway

Increased intracellular glucose levels overload ETC and are shunted into alternative pathways, in turn generating ROS. In the polyol pathway, accounting for >30% of glucose metabolism during hyperglycemia [54], glucose is converted by NADPH-dependent aldose-reductase to the sugar alcohol sorbitol, and sorbitol to fructose by sorbitol-dehydrogenase. The oxidative stress generated by these reactions depends on the consumption of NADPH, a cofactor required to regenerate the ROS scavenger glutathione (GSH), and on NAD+ reduction to NADH, which is subsequently oxidized by NADH oxidase, with consequent production of superoxide ions [55]. Aldose-reductase has been indeed implied in the increased expression of inflammatory cytokines [56,57].

#### 2.1.4. Generation of Advanced Glycation End-Products (AGEs)

In conditions of hyperglycemia, the nonenzymatic fragmentation of the glycolytic intermediate triose phosphate produces methylglyoxal, precursor of the majority of AGE products formed by a nonenzymatic reaction of either ketones or aldehydes and the amino groups of proteins, during which large amounts of ROS are generated [25].

AGEs can interact with two types of cell surface receptors, scavengers involved in AGE removal and receptors for AGE (RAGEs), which initiate detrimental cellular signals, promoting inflammation and atherogenesis [29,58,59]. As an example, AGEs dose-dependently activate oxidative stress-mediated P38 activation of mitogen-activated protein kinase (MAPK) signaling in endothelial cells, which enhances NO synthesis inhibition by AGEs [60].

Both AGEs and methylglyoxal also promote the expression of RAGEs ligands. In particular, oxidized AGEs activate RAGEs to stimulate NADPH oxidase (NOX) [61], another important source of ROS production. NOX, which in healthy state determines ROS production, in pathological conditions may be hyper-expressed and hyperactive, as observed in cultured mice microvascular endothelial cells (MMECs) and human umbilical artery endothelial cells (HUAECs) exposed to high glucose [62,63]. Cells exposed to glucose fluctuations produce higher levels of NOX-derived ROS as compared to cells steadily exposed to high glucose, thus indicating the detrimental effect on vascular health of acute glycemic variations [64].

#### 2.1.5. Activation of Protein Kinase C (PKC)

PKC is a serine/threonine related protein kinase acting in a wide variety of biological systems and regulating cell growth and proliferation, senescence, and apoptosis. The enzyme, once activated, induces many atherogenic processes, like ROS overproduction, endothelial dysfunction, increased vascular permeability, and inhibited angiogenesis [33,65].

In particular, NOX PKC-dependent activation is considered among the major sources of high glucose-induced ROS production, even more than mitochondrion [66,67].

In either a hyperglycemic or diabetic environment, PKC is activated by oxidative stress and AGEs and by diacylglycerol (DAG), whose levels increase in endothelial cells due to the shunting of glycolytic intermediates to dihydroxyacetone phosphate [65,68]. DAG-PKC is among the several cellular pathways activating when oxidative stress causes DNA fragmentation and stimulation of the DNA repair enzyme, nuclear poly ADP ribose polymerase (PARP). This enzyme inhibits the glyceraldehyde-3-phosphate dehydrogenase (GAPDH), shunting early glycolytic intermediates into pathogenic signaling pathways, including AGE, polyol, DAG-PKC, and hexosamine pathways [25].

### 2.2. Endothelial Apoptosis and Senescence

Endothelial cell apoptosis and senescence are pivotal processes for the development of atherosclerosis, due to their activation by a plethora of pathways sharing the common pathophysiological mechanism of oxidative stress [69,70,71].

Studies on cultured ECs have shown that the promotion of senescence features (e.g., shortening of telomere length, elevated DNA damage, increase genomic instability and growth arrest) can be modulated by two factors intrinsically related to diabetes, high glucose [72], and AGE products [73], thus enhancing the intracellular levels of oxidative stress [74,75,76]. The implied cellular signals are diverse. As observed in high glucose exposed umbilical vein endothelial cells (HUVECs), Bax protein expression increases in the absence of Bcl-2 modifications, producing an elevated Bax/Bcl-2 ratio which activates the cleavage of procaspase-3 into active caspase-3, a crucial mediator of apoptosis [77]. As well, also the high-glucose induced NF-kB-dependent activation of c-Jun N-terminal kinase (JNK) and ROS-dependent Akt dephosphorylation may be involved [78].

Intriguingly, carbonic anhydrase, overexpressed in endothelial cells of diabetic ischemic heart, determines endothelial cell apoptosis in vitro, thus playing a key role in the remodeling process [79].

### 2.3. Other Pathogenetic Mechanisms of Vascular Dysfunction

A dysregulation of microRNAs (miRNAs), small non-coding RNAs, may contribute to the progression of atherosclerosis and diabetes-induced vascular dysfunction. As an example, a reduction in miRNA-126 levels has been associated with an increased leucocyte adherence to ECs and impairment of peripheral angiogenesis in T2DM [80]. Moreover, miR-29c and miR-204 were significantly dysregulated in atherosclerotic plaques from patients with DM [81].

T2DM has been proven as characterized by an imbalance of gut microbiota, which can directly promote atherogenesis by oxidative stress, inflammation, and changes in some metabolites, even though the bacteria possibly associated with progression of diabetes-accelerated atherosclerosis have not been identified yet [29].

## 3. Metformin Promotes Cardiovascular Health

Targeting and reduction of ED, an earlier phenomenon among the vascular abnormalities induced by cardiovascular risk factors, may represent a way to slow down diabetes-associated macrovasculopathy.

Since hyperglycemia-induced ROS may be the factor primarily involved in endothelial damage in diabetes, a protective action for correction of oxidative stress could be predicted. However, intervention studies in humans using orally administered antioxidants such as vitamins E and C have not been proven effective [82].

On the other hand, any anti-hyperglycemic drug achieving a rigorous glycemic control should mitigate the deleterious impact of diabetes on endothelium. However, despite antihyperglycemic effectiveness, not all these agents are able to reduce the CVD risk. Some drugs have been reported as independently associated with an increased risk (e.g., heart failure for rosiglitazone) [83], whilst others, likely provided of additional pleiotropic actions, resulted protective for the cardiovascular system. In this context, metformin, GLP-1 agonists (GLP1RA) and SGLT2 inhibitors (SGLT2i) obtained the strongest evidence for a beneficial effect on the endothelial function [84]. GLP1RA and SGLT2i have been approved for diabetes therapy in the most recent years. Therefore, it does not surprise the larger data on cardiovascular benefits available only for metformin which, after a 60-years history supporting its use, remains the first-choice agent for most T2DM patients.

The ability of metformin to reduce the diabetes-related CV risk arises from direct effects on the endothelium regardless, at least to some extent, of an improvement in metabolic disturbances (i.e., insulin resistance and hyperglycemia), and commonly associated risk factors (i.e., dyslipidemia and hypertension) [85,86,87].

### 3.1. Overview on Metformin

#### 3.1.1. Historical Notes

Metformin (1,1-Dimethylbiguanide) is a synthetic derivative of galegine, a compound of French lilac tested as a glucose-lowering agent in humans in the 1920s, but soon discarded due to its toxicity [88,89]. Its anti-hyperglycemic effectiveness has been demonstrated more than half a century ago by the French medical doctor Jean Sterne and the drug has been first used the UK in 1958 under the trade name Glucophage R (‘glucose eater’). The Food and Drug Administration (FDA) approved it for T2DM treatment only in 1994, after 20 years of use in Europe [88].

Despite long history and large clinical experience, metformin mechanism of action still remains not fully understood and even controversial, as it often happens with drugs of herbal origins not primarily designed for a specific cellular target.

#### 3.1.2. Pharmacological Effects on Glucose Metabolism

Metformin primarily regulates glucose homeostasis. Specifically, it inhibits liver glucose production by the downregulation of hepatic gluconeogenesis and glycogenolysis. Metformin also alleviates IR, with an enhancement of peripheral glucose uptake via GLUT4 transport and subsequent significant reduction of plasma insulin levels [90,91]. Most recent evidence reports an important contribution about the beneficial metabolic responses to metformin before drug absorption, due to the interaction with gut microbiota and the modulation of incretin axis [92,93], thus supporting the role in the relationship between glycemic index and cardiometabolic diseases [94].

Over the past few decades, metformin has realistically emerged as a drug acting not only on specific targets of metabolism, though also on a series of other mechanisms and signaling pathways [95,96], some of which involved in the atherosclerosis prevention [97].

#### 3.1.3. Activation of AMPK

In the literature there is a general consensus about the key role of AMK activation on metformin’s cellular actions, in particular at level of liver and skeletal muscle [98]. AMPK is a heterotrimeric serine/threonine protein kinase containing one catalytic α subunit and two non-catalytic subunits, scaffold β and regulatory γ subunits. Each subunit has two isoforms (*α*1, *α*2, *β*1, *β*2, *γ*1, *γ*2, and *γ*3), widely expressed in different tissues and subcellular sites [99].

AMPK is a major regulator of cellular energy homeostasis coordinating the enzymes involved in carbohydrate and fat metabolism to enable ATP conservation and synthesis. Conditions of increased AMP:ATP ratio (exercise, metabolic stress, and hypoxia) activate AMPK, which switches off the ATP-consuming pathways and on the ATP-generating ones [98].

Increasing evidence suggests that the role of AMPK goes beyond energy metabolism control, as the enzyme may regulate a very wide range of cell functions accounting for a variety of metformin pleiotropic actions [100,101]. As an example, AMPK stimulates eNOS production [102], thus supporting a protective role of this kinase on the endothelium, as demonstrated in a study on obese rats [103,104].

The phosphorylation at Thr1172 of the *α*-subunit activates AMPK, whilst AMP and/or adenosine diphosphate (ADP) binding to the *γ*-subunit protects the enzyme against dephosphorylation [105]. Upon ATP depletion, AMPK is phosphorylated and activated by upstream kinases such as liver kinase B-1 (LKB1), constitutively expressed in most cell types [106], and calcium/calmodulin-dependent protein kinase-beta, activated by intracellular calcium and expressed only in certain cell types, including ECs [107,108]. Intriguingly, AMPK has been found dysregulated in experimental animal models and in humans with either metabolic syndrome or T2DM [109].

Metformin and AICAR (5-amino-imidazole carboxamide riboside) are the two most commonly used AMPK activators. AICAR is an analog of AMP directly activating the enzyme, but not suitable for human use [110]. On the contrary, metformin is not a specific activator of AMP, but it can be used in humans. How exactly metformin activates AMPK is still unclear [111]. The drug might increase the phosphorylation of AMPK catalytic α subunit at Thr1172, as reported by studies on primary hepatocytes [112], or inhibit AMP deaminase [113].

On the other side, there is large evidence that enhanced AMPK expression is secondary to the increased intracellular ADP/ATP and AMP/ADP ratios resulting from a mild, transient and specific inhibitory action of metformin on ETC’s mitochondrial complex I (NADH: ubiquinone oxidoreductase) [90,93]. Even this mechanism is debated and the extent to which it is physiologically relevant is still uncertain, as the required concentration seems about 500–1000 times than the highest attained therapeutically [114].

Beyond ETC’s complex I inhibition, other mitochondrial actions have been described, including a direct binding of metformin to mitochondrial copper ions [115] and a non-competitive inhibition of mitochondrial glycerol 3-phosphate dehydrogenase shuttle, producing impaired respiration, reduced cytoplasmic NAD+/NADH ratio and undermined glucose production from both glycerol and lactate [116]. The physiological relevance of these mechanisms is unclear. Incidentally, whether metformin can access mitochondria to a sufficiently high concentration to inhibit complex 1 or exert other actions is still object of debate [117].

Considering the plurality of cellular sites of metformin action, not all the effects of the drug are necessarily mediated via either the direct or indirect activation of AMPK. For instance, Foretz et al. reported that metformin was able to inhibit liver gluconeogenesis in transgenic mice lacking AMPK subunits and LKB1 [118], whilst Buse et al. showed that a significant component of the anti-hyperglycemic effects of metformin resided in microbiome [92].

### 3.2. Metformin Reduces Cardiovascular Mortality in Diabetes

Publication in 1998 of the United Kingdom Prospective Diabetes Study (UKPDS), a trial designed to assess whether intensive blood-glucose control reduced the risk of macrovascular or microvascular complications in T2DM patients, represented the event which has changed metformin’s history. Remarkably, UKPDS findings attributed to metformin the role of first choice anti-hyperglycemic drug after demonstrating, in overweight patients randomized to metformin as compared to conventional dietary measures, a risk reduction of 39% for nonfatal myocardial infarct, 42% for diabetes-related death, and 36% for all-cause mortality [119].

A Cochrane meta-analysis supports the benefits of metformin as compared to other antidiabetic drugs, proving a reduced all-causes mortality [120].

Two further meta-analysis have strengthened this result. The first showed metformin as the only antidiabetic agent able to improve all-cause mortality without causing any harm in diabetic patients with heart failure [121]. The other instead reported significantly lower all-cause mortality rates in diabetic individuals taking metformin as compared either to non-diabetics or diabetics receiving non-metformin therapies [122]. On the contrary, an evaluation of 35 clinical trials including over 18,000 participants found a significant benefit for metformin versus placebo/no therapy, but not versus active-comparators [123]. Another metanalysis of randomized trials has left doubts about whether metformin reduces risk of cardiovascular disease in T2DM or not [124]. Moreover, an observational study using the REACH Registry showed an association between metformin’s use in secondary prevention and a decreased mortality [86]. In a retrospective Danish cohort study on T2DM patients admitted with myocardial infarction and not treated with emergent percutaneous coronary intervention, monotherapy with sulfonylureas was associated with increased cardiovascular risk compared with metformin monotherapy [125].

## 4. Protective Properties of Metformin on Endothelium

Metformin displays multiple beneficial effects against CVD, among the most relevant those exerted on vascular endothelial function [8,114,117,126]. A 4.3-year clinical trial has shown a metformin-associated reduction of several plasma ED biomarkers (e.g., vWF, sVCAM-1, t-PA, PAI-1, and sICAM-1), regardless of changes in HbA1c, insulin dose, and body weight. The authors reported that ED improvement explained about 34% of the reduced cardiovascular risk associated with biguanide treatment [127].

The endothelial protection exerted by metformin may not represent the product of a single pharmacological action, though rather the result of concurrent multiple mechanisms involving endothelium-dependent vascular response, oxidative stress, leukocyte-endothelium interactions, mitochondrial function, and others. Literature data highlight the role of hyperglycemia in ED pathogenesis [128], even though metformin therapeutic concentrations may improve vascular endothelial reactivity in non-diabetic patients, regardless of glucose levels [129]. More likely, metformin exerts both anti-hyperglycemic-mediated and direct actions on endothelial function.

We will now discuss metformin’s impact on endothelium and possible underlying cellular and biochemical mechanisms observed in human investigations and in preclinical studies.

### 4.1. Metformin Improves Endothelium-Dependent Vascular Response

Almost 30 years ago, Marfella et al. demonstrated that metformin improved hemodynamic and rheological responses to infusion of l-arginine, the natural precursor of NO, in newly diagnosed T2DM patients without micro- and macrovascular complications [130].

At the dawn of the third millennium, when NO has begun to emerge as a protective CV factor [131], the analysis of vascular response to metformin in T2DM patients by a direct measurement with forearm strain-gauge plethysmography, proved an improvement of endothelium-dependent vasodilation after a 12-week treatment, indicating the endothelium as the primary site for dysfunctional blood flow. Notably, ED improvement has been associated with a reduction in whole-body IR [132].

#### 4.1.1. Role of Insulin Resistance Correction

Insulin is known to promote NO production by activating the PI3K/Akt/eNOS signaling pathway, which results in vasodilation and vascular protection [133,134].

Once IR develops, pathway-specific impairment in PI3K-dependent signaling may cause imbalance between production of NO and secretion of ET-1, thus leading to endothelial dysfunction [135].

**Figure 2:**
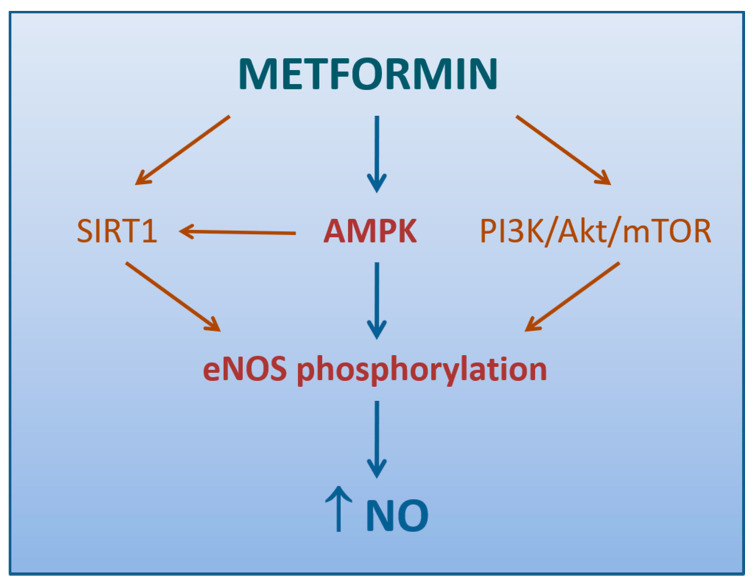
Mechanisms by which metformin promotes NO production (direct arrows indicate the direction of the pathway).

The aforementioned study by Mather et al. supported the conclusion that endothelium-dependent vascular response correction by metformin was more likely secondary to improved insulin signaling [132] (Figure 2), consistently with previous reports on these subjects [136]. Steinberg et al. had already demonstrated that excessive exposure of endothelium to free fatty acids (FFAs) increased O_2_^−^**·** production, impaired NO activity, and reduced endothelium-dependent vasodilation [137]. IR is characterized, along with the involvement of numerous other systems [138], by sustained elevations in serum FFAs and failure of appropriate suppression following meals, due to a compromised ability of insulin-resistant adipocytes to store and retain FFAs [139]. The link between FFA excess and ED may lie in a sequential process starting from the increased de novo synthesis of DAG, which activates PKC, in turn responsible for endothelial O_2_^−^**·** overproduction via NOX stimulation [67], eNOS inhibition [140], and activation of a vicious worsening of insulin signaling in the endothelial cells [141].

Preclinical studies in mesenteric arteries and aortas from insulin-resistant rats support an improvement of ACh-induced vasodilation by treatment with the insulin sensitizer metformin [142,143]. Indeed, the relationship between IR and ED in humans is not so clear. On the one hand, metformin improves endothelial function in non-diabetic insulin resistant populations [144]. Otherwise, troglitazone, a ligand of nuclear receptor peroxisome proliferator-activated receptor (PPAR)-γ with insulin-sensitizing actions, administered to obese subjects, determined an improvement in insulin sensitivity but no effects on both endothelium-dependent and independent vascular responses [145]. Accordingly, in a study on T2DM patients treated with sulfonylureas, the improvement in ED with the addition of either metformin or pioglitazone did not seem associated neither with a better glycemic control nor with insulin sensitivity [146]. Moreover, a pilot trial in uncomplicated T1DM patients showed a significant improvement of ED, irrespective of glycemic control and body weight, after a 6-months metformin treatment in add-on to basal-bolus insulin regimen [147].

#### 4.1.2. Role of AMPK Activation

An attractive hypothesis of how metformin enhances endothelium-dependent vasodilation may reside in the activation of AMPK [148]. It has been extensively demonstrated that several stimuli, not last metformin, may induce AMPK-dependent eNOS phosphorylation, thus resulting in increased NO production and vasodilation in conduit arteries [102,149,150] (Figure 2). Matsumoto and colleagues reported an improvement of the endothelium-dependent responses by metformin even in the resistance arteries of diabetic rats, thanks to the suppression of prostanoid signaling [151]. Later, a study on mice with endothelium-specific deficiency of α-catalytic subunit of AMPK, demonstrated eAMPK α1 as the main upstream enzyme that mediates EDH responses of microvessels, thus regulating blood pressure and coronary flow responses in vivo [152]. Since these findings have not been confirmed, the contribution of AMPK in the tone regulation at level of microvasculature, where EDH signaling plays a more prominent role, still remains controversial [153].

#### 4.1.3. Other Mechanisms

Based on the evidence that Sirtuin-1 (SIRT1), a NAD-dependent deacetylase with antiaging activities, enhances the activity of eNOS with NO generation and endothelial-dependent vascular relaxation [154], we can speculate that metformin indirectly increases eNOS activity by directly inducing SIRT1 expression and/or activation (Figure 2). This hypothesis is supported by the observation that a 72-h exposure to metformin may reduce hyperglycemia-induced endothelial senescence and apoptosis via a SIRT1-dependent process [155].

Ghosh et al. demonstrated that a brief exposure of aortic tissue and microvascular endothelial cells to metformin can either reverse or reduce the high glucose-induced ED via mechanisms linked to increased phosphorylation of eNOS and Akt, a cytosolic protein involved in the intracellular signaling pathway PI3K/Akt/mTOR regulating the cell cycle. Of note, this response was not accompanied by changes either in AMPK phosphorylation or SIRT1 expression [156].

### 4.2. Metformin Promotes Antioxidation

ROS are strongly involved in ED occurrence, due to their vasoconstrictor action and the reactivity with NO to produce ONOO^−^**·**, with further reduction of NO bioavailability [157].

Large evidence supports metformin inhibitory effect on oxidative stress, in vitro in hyperglycemic environments [158,159] as well as in vivo in high fructose-fed rats [160] and T2DM patients [161].

Experiments in BAECs and HUVECs in the presence of either NOX inhibitor apocynin or ETC inhibitor rotenone, report that metformin inhibits ROS formation from both respiratory mitochondrial chain and NOX [162,163]. PKC-NOX pathway inhibition by metformin was later confirmed in human aortic endothelial cells [164]. In rats exposed to the prooxidant rotenone, metformin’s co-treatment is able to correct redox imbalance and toxicity of erythrocytes [165]. Metformin has been also reported to prevent the rise in lipid peroxides and oxidized proteins and the fall of mitochondrial aconitase activity, a sensitive parameter for the mitochondrial generation of ROS inside in aortic tissue, heart and kidney of diabetic Goto-Kakizaki rats [166] and the DNA damage related to oxidative stress in lymphocytes from elderly subjects [167]. The significant reductions in NO release and the pronounced increase in nitroxidative stress observed in obese Zucker rats significantly reverted with metformin treatment, as a result of improved eNOS coupling and bioavailable NO, and other mechanisms regulating endothelial function beyond glucose control [168].

**Figure 3:**
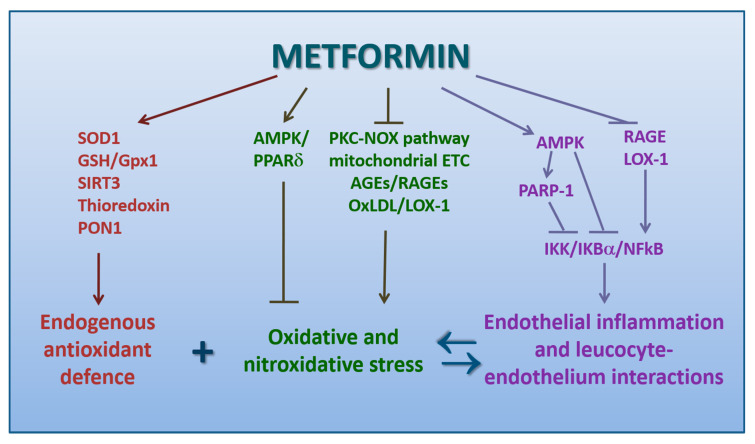
Schematic picture of the mechanisms by which metformin exerts a protective action against oxidative stress and endothelial dysfunction (direct arrows indicate the direction of the pathway, whilst blocked arrows stands for inhibition of that specific pathway. Double arrow stands instead for a bidirectional reaction).

The underlying mechanisms of these antioxidant properties of metformin still remain controversial (Figure 3). The scavenging direct capacity of trapping free radicals is negligible [169].

More likely, metformin enhances the endogenous antioxidant defense by preventing the hyperglycemia-related inhibition of glucose-6-phosphate-dehydrogenase (G6PDH), which would either hamper the regeneration of reduced GSH [170] or increase superoxide dismutase-1 [171]. AMPK pathway has been proven to potentially reduce the intracellular ROS level by activating the fork-head transcription factor 3 (FOXO3), subsequently upregulating thioredoxin expression, a major component of an important endogenous antioxidant system, which promotes the reduction of proteins by cysteine thiol-disulfide exchange [172]. This pathway seems responsible for the attenuation of intracellular ROS levels induced by metformin in primary human aortic endothelial cells exposed to palmitic acid [173].

Otherwise, metformin decreases ROS cellular production. Several experiments have proven metformin’s capacity to downregulate NOX, among the major cellular producers of ROS [174,175,176,177]. Accordingly, a study in cultured HUVEC and murine aortas isolated from AMPK-α2 deficient mice demonstrated that this enzyme acts as a physiological suppressor of NOX and ROS production in endothelial cells [178]. Since oxidative stress is proportional to the accumulation of AGEs in diabetic animals [179], the antioxidant activity of metformin may be partially due to the inhibition of glycation, a process directly related to free-radical production.

Other mechanisms may be overexpression of SIRT3, a NAD+-dependent deacetylase specifically located in the mitochondria, and glutathione peroxidase 1, which protects leukocytes against oxidative stress by reducing hydroperoxides [180,181]. Metformin may also inhibit endoplasmic reticulum stress and oxidative stress by activating AMPK/PPARδ pathway, as reported in a study on aortae from obese diabetic mice [182] (Figure 3).

### 4.3. Metformin Counteracts the Pro-Atherogenic Role of oxLDL and LOX-1

Oxidized low-density lipoprotein (OxLDL), as well as class E scavenger lectin-like oxidized receptor 1 (LOX-1) mediating OxLDL uptake by vascular cells, are involved in events critical in atherosclerosis development from ED until plaque instability and rupture [183]. OxLDL is a product of chronic oxidative stress which, in parallel, can act as pro-oxidant by stimulating NOX and ROS generation [184]. On the other hand, LOX-1 may bind with high affinity a broad spectrum of structurally distinct ligands besides OxLDL, among which AGEs which, in turn, upregulate LOX-1 expression in diabetes [185].

Metformin has been proven to inhibit the expression of both RAGEs and LOX-1, more likely through a modulation of redox-sensible nuclear factors, including NF-kB, which are involved in such receptor cell expression [186].

Exposition of cultured endothelial cells to oxidized and glycated LDL (HOG-LDL) causes aberrant ER stress via enhanced sarcoplasmic/endoplasmic reticulum Ca^2+^ ATPase oxidation, significantly mitigated by either pharmacological (included metformin) or genetic activation of AMPK, which results in an improved endothelium-dependent relaxation [187].

OxLDL signals mainly activate via LOX-1 diverse cellular second messengers, including NF-κB and AP-1, two oxidative stress-responsive transcription factors involved in the regulation of cytokines, chemokines, and adhesion molecules in endothelial cells. In turn, some of the induced cytokines activate NF-κB and AP-1, thus reinforcing the inflammatory signaling cascade [188].

A study on human primary coronary artery endothelial cells showed for the first time that OxLDL induced ED, cell death, and impaired vasorelaxation, partially via TRAF3IP2, a redox-sensitive cytoplasmic adapter protein and an upstream regulator of IKK/NF-κB and JNK/AP-1. Moreover, while native HDL3 inhibited, oxidatively-modified HDL3 potentiated OxLDL-induced TRAF3IP2 expression. AMPK activators (adiponectin, AICAR and metformin), through AMPK-dependent Akt activation, antagonized the pro-apoptotic effects of OxLDL-induced TRAF3IP2 expression [189].

A study on HUVECs showed that SIRT1 and AMPK silencing decreased the protective function of metformin against OxLDL-increased LOX-1 expression and OxLDL-collapsed AKT/eNOS levels [190].

Both in diabetic rats [191], and newly diagnosed diabetic patients [192], metformin has been shown to restore the activity of paraoxonase 1, an antioxidant associated with circulating HDL that hydrolyzes lipid peroxides in LDL.

### 4.4. Metformin Inhibits Endothelial Inflammation and Leukocyte-Endothelium Interactions

Beyond its anti-oxidative properties, metformin also exerts eminent anti-inflammatory effects, as expected from an AMPK activator [193].

In a study published in 2003, treatment of human ECs with AGEs for up to 12 h has been shown to significantly increase human monocyte adhesion, an effect prevented by the presence of metformin in incubation medium [194]. Incidentally, the drug also prevented monocyte differentiation into macrophages and foam cell, a process that metformin regulates via AMPK-mediated inhibition of STAT3 activation [195].

These findings were later extended. In fact, metformin has been demonstrated to suppress the cytokine-induced activation of NF-kB in HUVECs. As a consequence, NF-kB-regulated gene expression of various inflammatory and cell adhesion molecules was inhibited. This effect was determined via the AMPK-dependent inhibition of the IKK/IKBα/NF-KB pathway [196].

An excessive and sustained oxidative stress can cause overactivation of poly (ADP-ribose) polymerase-1 (PARP-1), which worsens the oxidative stress and stimulates pro-inflammatory and necrotic responses [197]. An investigation on HUVECs and in vivo on mice has demonstrated a possible metformin involvement in a pathway linking AMPK, PARP-1, and B-cell lymphoma–6 protein (Bcl-6) in the prevention of monocyte adhesion to endothelial cells and attenuation of endothelial inflammation. PARP-1 binding to Bcl-6 intron 1 has been proven to suppress the transcription of Bcl-6, a corepressor for inflammatory mediators recruiting monocytes to vascular endothelial cells upon inflammation. Conversely, phosphorylation of PARP-1 at Ser-177 by activated-AMPK decreased its binding to Bcl-6 intron 1, with subsequent transcriptional up-regulation of Bcl-6 and co-repression of VCAM-1, MCP-1, and MCP-3 to finally result in an anti-inflammatory phenotype [198]. A later report further confirmed that vascular protection of metformin partially occurs through the activation of the AMPK-PARP-1 cascade [199].

In a study on retinal endothelial cells under hyperglycemic conditions, SIRT1 activation by metformin significantly attenuated ROS mediated activation of PARP through the upregulation of LKB1/AMPK, with the subsequent suppression of NF-kB, as well as of proapoptotic gene Bax [200]. All these mechanisms are summarized in Figure 3.

### 4.5. Metformin Attenuates the Apoptosis, Senescence, and Death of Endothelial Cells

Mitochondria are the powerhouse of the cell, providing over 90% of ATP consumed by the cell, but they also play an important role in the commitment to cell death [201]. Several intermembrane space proteins have no pro-apoptotic activity when persisting inside mitochondria, though they promote cell death once released into the cytosol by opening an inner membrane channel, the so-called permeability transition pore (PTP) [202]. Metformin has been found to prevent the PTP opening determined by the high glucose-induced oxidative stress in several endothelial cell types [203], and the biguanide given at the time of reperfusion may reduce myocardial infarct size in the heart of both non-diabetic and diabetic rats [204].

**Figure 4:**
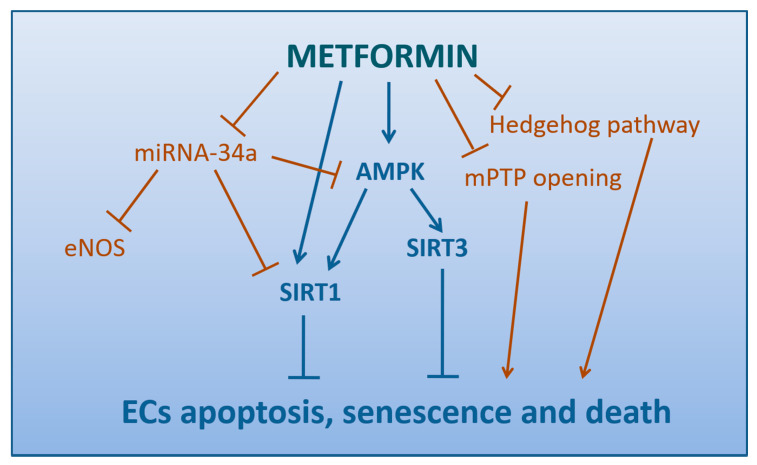
Mechanisms by which Metformin protect Endothelial Cells from apoptosis, senescence, and death (direct arrows indicate the direction of the pathway, whilst blocked arrows stands for inhibition of that specific pathway).

SIRT1 plays a central role in the regulation of endothelial cell growth, senescence, and apoptosis, as well as in atherosclerosis development [205]. Metformin may be considered either a direct or LKB-1/AMPK-mediated modulator of SIRT1 expression, able to alleviate hyperglycemia-caused endothelial senescence and cell death (Figure 4). Similarly to the results by Zheng et al. (see Section 4.4) [200], in a study on MMECs hyperglycemia has been proven to accelerate endothelial apoptosis and senescence via changes in SIRT1 expression and downstream signaling targets FoxO-1/p53, whereas metformin prevents these detrimental effects attenuating hyperglycemia-induced oxidative stress and upregulating SIRT1 expression [206].

miRNA-34a has been reported as highly expressed in ECs and it may directly bind to SIRT1, the so-called anti-ageing gene, thus inhibiting sirtuin1 expression and regulating apoptosis via the sirtuin1-p53 pathway [207]. In HUVECs, miRNA-34a overexpression down-regulates sirtuin1 expression and induces ECs senescence, whereas miRNA-34a knock-down enhances sirtuin1 expression and attenuates endothelial senescence [208]. A study in MMCs has reported that hyperglycemia-mediated induction of miRNA-34a results in impaired angiogenesis, a defect revertible by therapeutic intervention with metformin, likely through the modulation of miRNA-34a levels which, in turn, regulates sirtuin1, AMPK and eNOS activity [206].

Using a H_2_O_2_-induced senescence model of human and murine fibroblast and HUVECs, autophagic dysfunction and decline in NAD+ synthesis have been shown as two features of senescent cells induced by oxidative stress, both restored by metformin through AMPK activation [209].

Two recent investigations have identified novel molecular mechanisms for metformin-mediated age-delaying effects on endothelium (Figure 4). The first is AMPK-mediated and lies on the regulation of mitochondrial biogenesis/function and senescence by H3K79me acting through SIRT3 [210]. Indeed, the second is AMPK-independent and consists in the downregulation of autophagy via the Hedgehog pathway, a signaling critically involved in adult tissue maintenance, renewal, and regeneration [211].

### 4.6. Metformin Inhibits Mitochondrial Fission

Mitochondria form a complex and dynamic network undergoing continuous cycles of fusion and fission events which are crucial to maintain organelle homeostasis [212]. Mitochondrial fusion seems beneficial as it distributes metabolites, proteins, and DNA throughout the mitochondrial population. In contrast, excessive mitochondrial fission may be detrimental due to accumulation of fragmented mitochondria with ETC impairment and mitochondrial ROS increase, as it occurs after cell exposure to high glucose concentrations [213]. In endothelial cells, mitochondrial fission contributes to the reduction in eNOS-derived NO bioavailability [214], impairment of angiogenesis [215], and induction of apoptosis [216]. An increased mitochondrial fission has been reported in different tissues of T2DM patients, more remarked in those with poor glycemic control [217,218].

AMPK activation by metformin may slow atherosclerosis development in diabetes by reducing the mitochondrial fission and its detrimental consequences. Using streptozotocin (STZ)-induced diabetic ApoE2/2 mice, a well-established model for the study of human atherosclerosis metformin has be found to reduce dynamin-related protein 1 (Drp1) expression and Drp1-mediated mitochondrial fission in an AMPK-dependent manner. Concomitantly, mitochondrial-derived superoxide release was mitigated, endothelial-dependent vasodilation improved, vascular inflammation inhibited, and atherosclerotic lesions suppressed [219].

Mitochondrial biogenesis is a response of stress adaptation to improve efficiency of cellular energy and preserve the cellular integrity [220]. The process has been frequently associated with the activation of AMPK by not well-defined mechanisms. A study by Le et al. has demonstrated that AICAR in endothelial cells induces mitochondrial biogenesis and stress adaptation via an AMPK/eNOS/mTORC1 pathway [221].

### 4.7. Other Protective Vascular Actions by Metformin

Several studies have shown that Ang II binding to Ang II type 1 receptor (AT1R) is involved in the progression of cardiovascular diseases, including atherosclerosis, hypertension, cardiac hypertrophy, and heart failure. Metformin has been shown to potentially decrease AT1R expression in mice aortas and attenuate vascular senescence and atherosclerosis induced by a high-fat diet, thus suggesting that AT1R downregulation may, at least partially, mediate the protective effect of metformin in the vascular system [171].

It has been further reported that AMPK pharmacological activation with metformin (as well as salicylate, resveratrol, and AICAR), inhibited inflammation in perivascular adipose tissue and improved ED against inflammatory insult in an AMPK/SIRT1-interdependent manner [222].

The loss of glycocalyx, a proteoglycan-rich hydrogel which separates blood from endothelium, represents an early event in the development of endothelial dysfunction [223]. A study demonstrated that metformin’s treatment, preserving glycocalix may restore the blunted hyperemic response in myocardial microvascular perfusion in rats challenged with a high-fat diet [224].

The proliferation and migration of human aortal smooth muscle cells, a well-known etiological factor of atherosclerosis, restenosis, and pulmonary hypertension, can be significantly inhibited by metformin through AMPK activation, even though this result has been obtained at very high drug concentrations, precluded to achieve in vivo [225].

The endothelial-to-mesenchymal transition (EndoMT), a cellular process involved in ED and vascular disease pathogenesis, is characterized by the loss of endothelial features and gaining of mesenchymal ones by ECs [226]. A study on HUVECs described that high glucose could induce EndoMT and suppress the endothelial protective axis of Kruppel-like factor 4 (KLF4), a master transcription factor maintaining vascular homeostasis, and Ch25h, a promoter of reverse cholesterol efflux. Metformin inhibited these effects by increasing Ch25h expression not only through KLF4, though also epigenetic changes, including DNA methylation and active histone modification [227].

## 5. Conclusions

Diabetes is a serious and global health problem affecting about 500 million people worldwide, a number expected to grow along with the associated high burden of premature and accelerated atherosclerosis impact on both life quality and expectancy. Since cardiovascular mortality is a major cause of death among individuals with T2DM, the prevention of macroangiopathies by preserving endothelial function represents a major therapeutic concern in this population.

Over its 60-year old history of use, multiple advantages of metformin have been proven, being inexpensive, mildly weight-lowering, relatively free of side effects other than gastrointestinal-related, with a very low risk of hypoglycemia and especially of so feared lactic acidosis [228]. Above all, an extensive pre-clinical and clinical literature details its vascular benefits.

Metformin as first choice treatment for T2DM patients is currently the most widely prescribed oral anti-hyperglycemic agent worldwide, with nearly 150 million annual prescriptions [229,230].

A US study has calculated that approximately 1 of 12 adults has a combination of pre-diabetes and risk factors which may justify the introduction of metformin as indicated by the American Diabetes Association guidelines [231]. Therefore, a higher proportion of relatively healthy individuals might benefit from metformin’s treatment to either prevent or delay both diabetes and cardiovascular events, even in secondary prevention, as recently demonstrated by a prospective study with a 24-month follow-up in pre-DM patients with stable angina and nonobstructive coronary stenosis [232].

However, a much wider use of this drug can be implemented as a viable cardiovascular preventive strategy, starting with the many millions of non-diabetic insulin resistant individuals with metabolic syndrome, until considering even the elderly population with its burden of several comorbidities [228,233], and those suffering from some common rheumatologic diseases closely associated with cardiovascular risk such as rheumatoid arthritis and gout [234].

The wide range of possible indications and the well documented benefits associated with its use fully deserve to metformin the attribute of “wonder drug” or “aspirin” of current times recently coined.
